# Improving the efficiency of crossbred Pradu Hang Dam chicken production for meat consumption using cold plasma technology on eggs

**DOI:** 10.1038/s41598-023-29471-6

**Published:** 2023-02-17

**Authors:** Apichaya Sakulthai, Choncharoen Sawangrat, Duangporn Pichpol, Jutamart Kongkapan, Tiranun Srikanchai, Rangsun Charoensook, Phanumas Sojithamporn, Dheerawan Boonyawan

**Affiliations:** 1 Department of Agro-Industry Technology Management, Panyapiwat Institute of Management, Nonthaburi, 11120 Thailand; 2 Department of Industrial Engineering, Faculty of Engineering, Chiang Mai University, Chiang Mai, 50200 Thailand; 3 Agriculture and Bio Plasma Technology Center (ABPlas), Thai-Korean Research Collaboration Center (TKRCC), Science and Technology Park, Chiang Mai University, Chiang Mai, 50200 Thailand; 4 Department of Veterinary Biosciences and Veterinary Public Health, Chiang Mai University, Chiang Mai, 50200 Thailand; 5 Department of Agricultural Sciences, Faculty of Agriculture Natural Resources and Environment, Naresuan University, Phisanulok, 65000 Thailand; 6 Department of Physics and Materials Science, Chiang Mai University, Chiang Mai, 50200 Thailand

**Keywords:** Zoology, Energy science and technology, Engineering, Physics

## Abstract

The Pradu Hang Dam chicken, a Thai Native Chicken (TNCs) breed, plays an important role in many regions of Thailand because of its chewiness. However, there are some challenges with Thai Native Chicken, such as low production and slow growth rates. Therefore, this research investigates the efficiency of cold plasma technology in enhancing the production and growth rates of TNCs. First, this paper presents the embryonic development and hatch of fertile (HoF) values of treated fertilized eggs. Chicken performance indices, such as feed intake, average daily gain (ADG), feed conversion ratio (FCR), and serum growth hormone measurement, were calculated to assess chicken development. Furthermore, the potential of cost reduction was evaluated by calculating return over feed cost (ROFC). Finally, the quality aspects of chicken breast meat, including color, pH value, weight loss, cooking loss, shear force, and texture profile analysis, were investigated to evaluate cold plasma technology's impact on chicken meat. The results demonstrated that the production rate of male Pradu Hang Dam chickens (53.20%) was higher than females (46.80%). Moreover, cold plasma technology did not significantly affect chicken meat quality. According to the average return over feed cost calculation, the livestock industry could reduce feeding costs by approximately 17.42% in male chickens. Therefore, cold plasma technology is beneficial to the poultry industry to improve production and growth rates and reduce costs while being safe and environmentally friendly.

## Introduction

As the world population grows yearly and reached 7.7 billion worldwide in 2019, food demand should increase with population growth. However, consumption has been growing faster than that of the population^1^. Poultry (i.e., chicken meat) is white meat that contains more nutrients than red meat (i.e., pork or beef), and there are no restrictions related to religion^2^. According to the EU agricultural markets briefs^1^, the consumption of chicken meat has increased significantly in all regions compared to other kinds of meat, such as beef, because of its low cost and convenience. Thailand is the worlds’ 5th biggest exporter of poultry^3^. Broilers, crossbreds, and native breeds are the three breeds available in Thailand^2^. Recently, the breeding goals to improve chicken have focused on morphological selection, physical properties, and production performance^4^. Although broiler chickens have better growth performance, feed efficiency, and meat yield than Thai Native Chicken (TNCs), the native chicken contains nutrients and bioactive compounds beneficial to human health and well-being^5^. Additionally, Thai consumers prefer native chicken because its meat is chewy, tasty, and of high health value^6^. Pradu Hang Dam, a TNCs, has black tail and body feathers mixed with red feathers on the top of the body, a red and black face, and a red pea comb.

The production of meat from TNCs is decreasing^7^. However, the production performance of TNCs, including Pradu Hang Dam, can be improved by selection, crossbreeding, or both, but improvement through selection requires a more extended period. Although improvement by genetic selection is faster, it requires native chickens with higher production potential^8^. In general, the male Pradu Hang Dam chicken is heavier than the females, and Pradu Hang Dam hens can produce approximately 95 to 175 eggs per year^9^. Supplemental feeding is another effective tool for transitioning from subsistence to economically viable semi-commercial production.

Many tools are used to enhance the growth performance of chicken, including antibiotics^10^, probiotics, or nanoparticles. Antibiotics have been applied in poultry feed as growth promoters and have shown significant advances since 1950. However, many countries are concerned about developing bacterial resistant populations and disturbing indigenous gut flora and banned the use^11^. Probiotics are considered alternatives for supplemental feeding and can be defined as a microbial feed supplement^12^ indicated that probiotics enhanced feed intake, growth performance, meat quality, egg production, and egg quality. In 2015, Olnood et al.^13^ illustrated that *Lactobacillus* spp. added to the feed did not improve weight gain, feed intake, or feed conversion rate (FCR) of broiler chickens during a 6-week experiment but improved anaerobic bacteria in the ileum and ceca and the number of lactic acid bacteria. Moreover, the effects of five dietary supplementations including Avilamycin (AM), *protexin* (PT), *Nutracid Focus* (NF), *Mannanoligosaccharides* (MOS), and *Vitacel* (VC), on humoral immunity, growth performance, mortality, and feed intake in Broiler chicken were compared^14^. The results showed that growth promoters significantly increased weight gain and the highest body weight gain in broiler chickens when protexin was applied. Finally, nanocomplexes^15^, gold nanoparticles^16^, and nanocurcumin^10^ have been used as dietary supplements to improve growth performance in broiler chickens.

A novel technology called plasma technology has been used to improve the growth performance of chickens. Plasma is the fourth state of matter, consisting of charged particles, excited radicals, reactive atoms, and molecules emitting electromagnetic radiation, including infrared, visible, and ultraviolet photons, and radiating transient electric fields^17^. In general, plasma is produced by applying an electrical field to a gas or a gas mixture^18^. The composition and characteristics of cold plasma were determined by varying the input parameters such as energy input, input power, gas ratio, gas pressure, gas flow rate, and radiation type of the electric field^19^. Because the composition and temperature can be adjusted, cold plasma has biological and biomedical applications, and it is safe for bio-objects^20^. The effects of argon plasma on the growth of soybean sprouts and the regulatory mechanism of energy metabolism were studied^21^. Although cold plasma has great potential advantages in various industrial fields such as surface treatment^22^, seed germination^23,24^, wounds healing^25^, etc., the investigation of cold plasma applications in the poultry industry is still in its early stages^26^.

Plasma was applied to eggs as a decontamination treatment against pathogens like *Salmonella*^27–29^. However, studies on the effect of cold plasma on chicken growth performance are limited and have not been studied in crossbred Pradu Hang Dam chickens. Therefore, this study aimed to determine the optimal conditions for cold plasma technology on crossbred, fertilized Pradu Hang Dam eggs incubated for four days to improve growth performance.

## Results and discussion

### Physical characterization of cold plasma

ROS/RNS concentrations of 2–5 ppm H_2_O_2_ were detected when using 100 ppm nitrate in 40 ml DI water at 5 min plasma exposure. According to the literature, ozone can be generated when ambient air is used as the working gas^30^. However, ozone is relatively unstable, has low solubility, and has a short half-life (~ 7–10 min)^30,31^. In contrast, the half-life of H_2_O_2_ is relatively longer (~ 10–20 min)^32^. Although the oxidation potentials of ozone and H_2_O_2_ were similar (~ 1.8–2.1 V)^33^, H_2_O_2_ may be expected to contribute more in this case because the plasma exposure time was increased to 30 s.

### Optimization of cold plasma generator for egg before hatching

**Figure 1:**
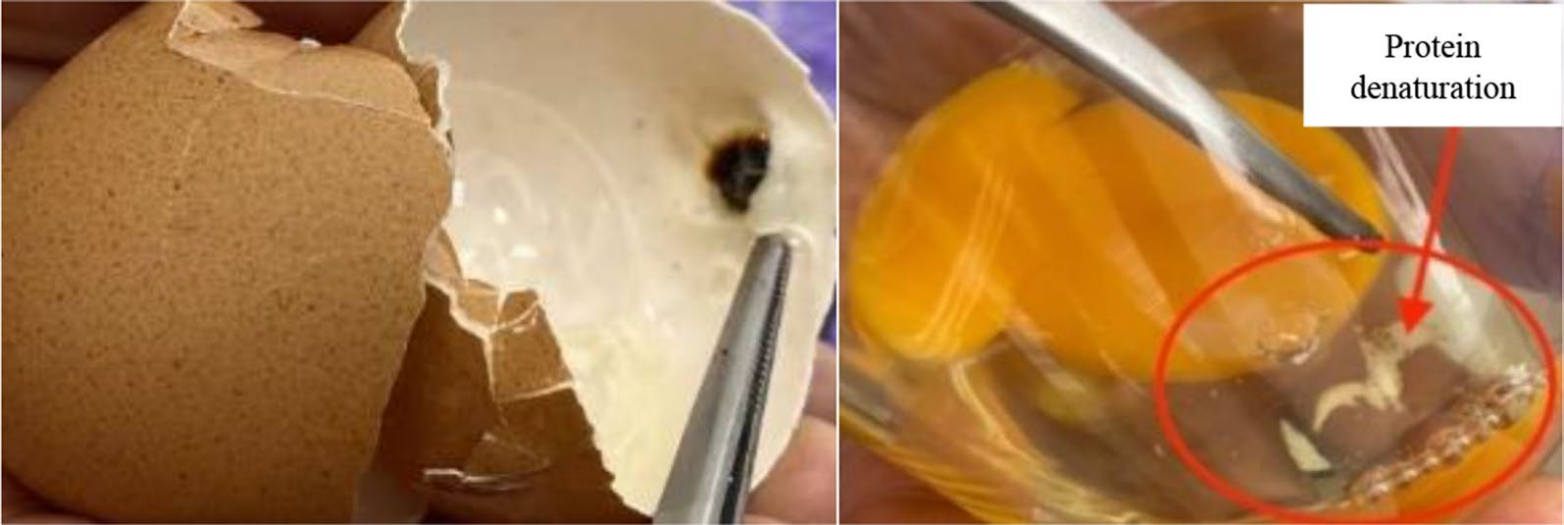
Burn mark on eggshell and protein denaturation in egg white.

The temperature laser was placed on the eggshell to measure the temperature change before and after plasma treatment. The results showed burn marks on the eggshell and protein denaturation in egg whites when the plasma exposure time was 30 and 60 s and the gap between the base and probe was less than 7 cm (Fig. 1). However, eggs treated with plasma for 30 s and a gap of 9 cm between the probe and base resulted in a temperature change of 0.3 ºC, but there was no effect on the physical or chemical properties of the eggs. When the plasma exposure time was 60 s and the gap between the probe and base was 9 cm, the liquid inside the egg vibrated, which may affect embryo development. Zhang, et al.^34^ indicated that discontinuous plasma exposure is due to the oval shape of the eggshell. Therefore, the results of this experiment indicate that plasma treatment parameters should be optimized before implementation.

### Efficiency of embryonic development and hatch of fertile

The hatch of fertile percentage and quality of day-old chick (DOC) were evaluated. The average of HoF (69.22%) and the number of good DOCs did not differ among treatments. However, the percentage of DOC differed between males (53.20%) and females (46.80%). A good DOC is free from physical disorders, injuries, and disabilities and shows the following characteristics: strong, standing on its legs, round eyes, well-closed belly, and invisible navel.

Thirteen chicks in each group were separated by sex. On days 0, 21, 42, and 63, the chicks were weighed, and the shank length was measured. The body weight of the DOC did not differ significantly among the treatments. The shank length of T30 was the highest among all treatments (Table 1). In male DOC, the shank length of T30 (2.12 ± 0.13) was significantly higher than the control (2.06 ± 0.10) and T20 (2.03 ± 0.10) (p-value < 0.05) but not significantly different from T10 (2.07 ± 0.11) (p-value > 0.05). There was no difference in body weight. In females, only the shank length of DOC in T20 was significantly higher than that of males.

**Table 1:** Shank length (cm) of Pradu Hang Dam chickens among treatments.

Age of chicken (day)	Gender	Treatments (mean ± SD)			
		0 s	10 s	20 s	30 s
DOC	Male	2.06 ± 0.10^b^	2.07 ± 0.11^ab^	2.03 ± 0.10^b,y^	2.12 ± 0.13^a^
	Female	2.06 ± 0.09^b^	2.06 ± 0.12^b^	2.11 ± 0.15^ab,x^	2.13 ± 0.11^a^
	Total	2.06 ± 0.10^b^	2.06 ± 0.11^b^	2.07 ± 0.13^b^	2.12 ± 0.12^a^
21	Male	4.74 ± 0.24^c^	4.88 ± 0.23^b^	5.02 ± 0.26^ab,x^	5.10 ± 0.18^a,x^
	Female	4.95 ± 0.39^a^	4.76 ± 0.22^ab^	4.65 ± 0.16^b,y^	4.59 ± 0.21^b,y^
	Total	4.85 ± 0.34	4.82 ± 0.23	4.83 ± 0.28	4.85 ± 0.32
42	Male	6.18 ± 0.43^x^	6.36 ± 0.27^x^	6.25 ± 0.37^x^	6.41 ± 0.25^x^
	Female	5.68 ± 0.35^b,y^	5.98 ± 0.27^a,y^	5.75 ± 0.25^ab,y^	5.93 ± 0.29^a,y^
	Total	5.90 ± 0.46 ^b^	6.17 ± 0.33^a^	5.99 ± 0.40^ab^	6.17 ± 0.36^a^
63	Male	8.08 ± 0.40^x^	8.17 ± 0.25^x^	7.97 ± 0.48^x^	8.09 ± 0.39^x^
	Female	7.61 ± 0.29^y^	7.36 ± 0.43^y^	7.30 ± 0.25^y^	7.47 ± 0.24^y^
	Total	7.84 ± 0.42	7.77 ± 0.54	7.63 ± 0.51	7.78 ± 0.44

^a,b,c^ and ^x,y^, values with different superscript letters in a column and row, respectively, are significantly different (p-value < 0.05).

Moreover, the shank length of males in each treatment was greater than that of females at 21, 42, and 63 days of age (Table 1). In males, the shank lengths of T20 (5.02 ± 0.26) and T30 (5.10 ± 0.18) at 21 days of age were significantly higher than those at T0 (4.74 ± 0.24) and T10 (4.88 ± 0.23). However, there was no significant difference among treatments at 42 and 63 days. In females, the shank lengths of T0 (4.95 ± 0.39) and T10 (4.76 ± 0.22) at 21 days of age were significantly higher than those of T20 (4.65 ± 0.16) and T30 (4.59 ± 0.21). However, the shank length of T0 (5.68 ± 0.35) at 42 days of age was significantly lower than that of T10 (5.98 ± 0.27), T20 (5.75 ± 0.25), and T30 (5.93 ± 0.29).

**Figure 2:**
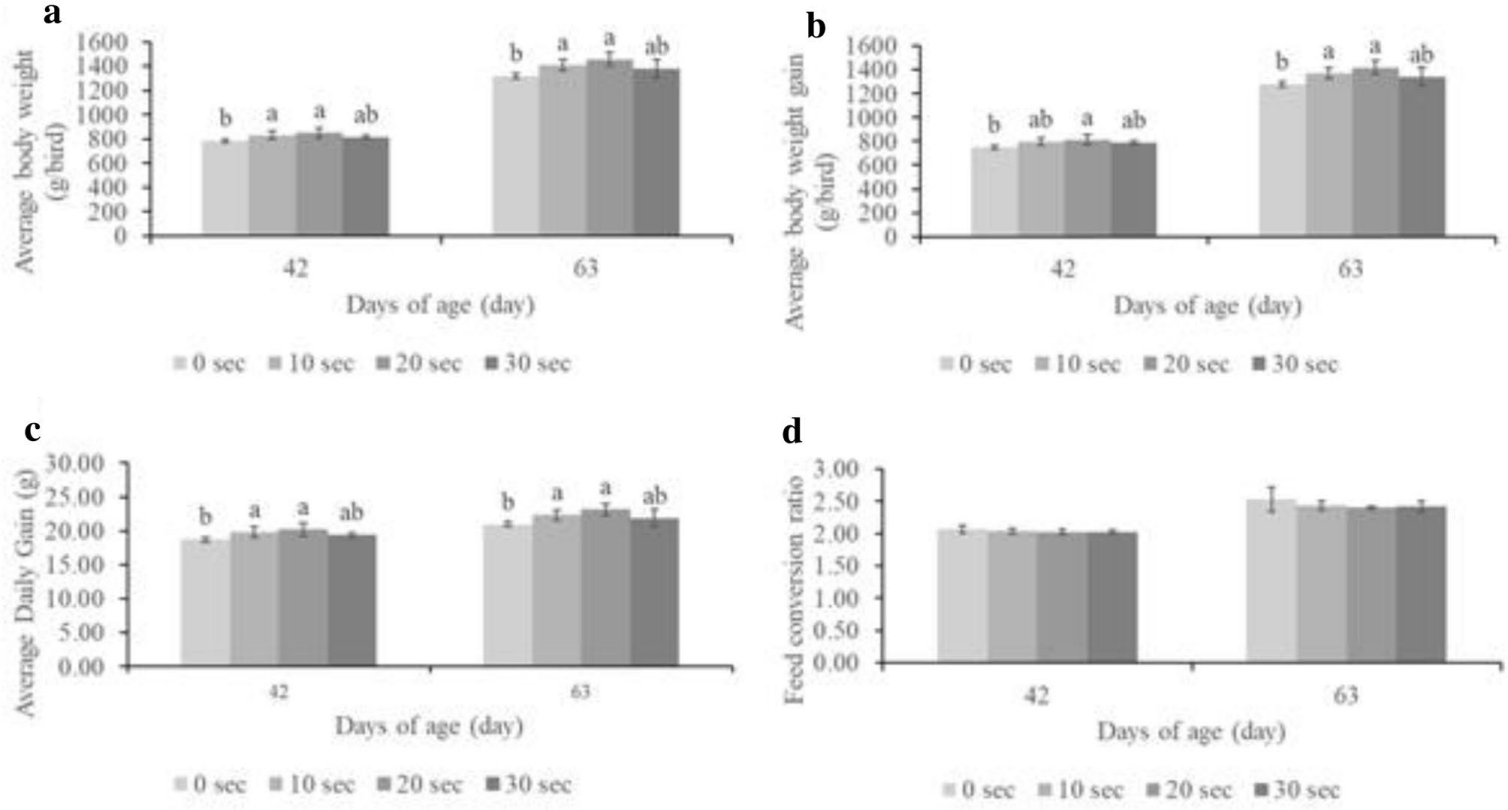
Male performance indices among treatments during 42 and 63 days. (**a**) Average body weight; (**b**) average body weight gain (**c**) average daily gain; (**d**) Feed conversion ratio. ^a,b,c^Values with different superscript letters are significantly different (p-value < 0.05).

Chicken performance indices, including feed intake, average daily gain (ADG), and feed conversion ratio (FCR), were calculated. The female chicken performance indices were not significantly different among treatments until 63 days. However, male body weight, ADG, and FCR were significantly different among treatments at 42 and 63 days (Fig. 2).

### Return over feed cost (ROFC)

The average ROFC (Baht/chicken) of male and female chickens under different treatment groups was calculated and is shown in Tables 2 and 3, respectively. For male chickens, the income from selling chickens was significantly higher in T20 (p-value < 0.05). However, the income from selling female chickens was not significantly different between the treatment groups. The feed cost during the experimental period (0–63 days) of male and female chickens was not significantly different between the treatment groups. Additionally, ROFC of male chickens (%/chicken) in T20 (17.42%) was the highest, followed by T10 (10.53%) and T30 (10.42%), when compared with T0. The ROFC of the female chickens was not significantly different from that of the control (T0) group. The present study confirmed that plasma-treated eggs have a greater effect on male chickens and is strong evidence that plasma technology can improve the livestock industries’ profit.

**Table 2:** The ROFC (Baht/chicken) of Male chicken under different treatment groups.

Particulars	T0	T10	T20	T30
Income from chicken sold (Baht/chicken)	79.198^d^ ± 1.324	84.556^b,c^ ± 2.814	87.542^a^ ± 3.395	82.858^b,c^ ± 4.674
Feed cost (Baht/chicken)	38.834 ± 2.588	39.942 ± 2.114	40.146 ± 1.227	38.286 ± 0.930
ROFC (Baht/chicken)	40.364^d^ ± 2.771	44.613^b,c^ ± 1.625	47.395^a^ ± 2.210	44.572^b,c^ ± 3.820
ROFC (%/chicken)	0.00	10.53	17.42	10.42

*ROFC* Return over feed cost, means within a row with different superscript differ significantly (p-value < 0.05).

**Table 3:** The ROFC (Baht/chicken) of female chicken under different treatment groups.

Particulars	T0	T10	T20	T30
Income from chicken sold (Baht/chicken)	67.627 ± 1.449	69.646 ± 1.999	69.185 ± 3.466	68.088 ± 2.040
Feed cost (Baht/chicken)	32.710 ± 1.259	33.044 ± 1.518	32.632 ± 1.721	32.214 ± 1.416
ROFC (Baht/chicken)	34.918 ± 1.685	36.602 ± 0.769	36.552 ± 1.753	35.875 ± 1.332
ROFC (%/chicken)	0.00	4.82	4.68	2.74

*ROFC* Return over feed cost, means within a row with different superscript differ significantly (p-value < 0.05).

### Serum growth hormone measurement

The levels of somatotropin (ng/ml) in the serum of male and female chicken at 21- and 42-day-old were detected. Although there were no significant differences among the treatments, T30 was the highest in males and females (Table 4). The somatotropin serum of 63-day-old chickens was less than 0.03 ng/ml.

**Table 4:** Comparison serum growth hormone level (Mean ± SD) among 4 treatments.

Age of chicken (day)	Gender	Treatments (mean ± SD)			
		0 s	10 s	20 s	30 s
21	Male	0.025 ± 0.033	0.021 ± 0.026	0.018 ± 0.035	0.121 ± 0.194
	Female	0.044 ± 0.033	0.028 ± 0.033	0.038 ± 0.044	0.054 ± 0.048
42	Male	0.097 ± 0.045	0.140 ± 0.041	0.198 ± 0.145	0.291 ± 0.217
	Female	0.152 ± 0.023	0.197 ± 0.050	0.888 ± 1.418	0.793 ± 1.265
63	Male	ND	ND	ND	ND
	Female	ND	ND	ND	ND

Not detected. (ND) when the result was less than 0.03 ng/ml.

In a previous study, plasma exposed at 11.7 kV for 60 s improved chicken embryo growth^35^. However, the duration and intensity of plasma exposure should be optimized for chickens in the embryonic stage. In this study, fertilized eggs were exposed with plasma at 6–10 kV for 30 s after 4 days of incubation. The eggs showed the highest HoF rate and shank length in the growing period of crossbred Pradu Hang Dam chickens, and the eggshell temperature was not affected. This confirmed that low-intensity plasma exposure improved cell proliferation^36^. However, an extended duration of plasma exposure induced cell death and apoptotic cells and decreased cell viability^37–39^.

This study revealed that the rate of hatching of fertilized eggs was higher in males than in females in the control and treatment groups. The treatment groups, especially T20, had a lower FCR. Although the shank length on the day of slaughter was not different in the plasma-treated group, body weight (BW) and ADG were higher in the treatment groups. This is expected to lower the feed conversion ratio while increasing exposure time. Moreover, plasma-treated male chickens grew significantly taller than plasma-treated female chickens. The results showed that plasma exposure had a greater effect on males, similar to the findings of Zhang et al.^40^. Moreover, the mRNA expression of plasma-exposed eggs increased^34^.

### Chicken carcass traits

The weight of slaughtered males was higher than that of females but similar among treatments (Table 5). In general, male chickens have a greater body weight than females. However, consumers prefer to have native chicken carcasses of small size because the bigger size would be tougher. The carcass percentage was not significantly different among the treatments. However, the percentages of retail cuts in terms of wing and shank of males in T10 (12.07 ± 0.41% and 5.94 ± 0.41%, respectively) were higher than the other groups (p-value < 0.05).

**Table 5:** Live and hot carcass weights of crossbred Pradu Hang Dam chickens among four treatments.

Items	Gender	Treatments (mean ± SD)			
		0 s	10 s	20 s	30 s
Live weight (g)	Male	1422.50 ± 51.95^x^	1417.94 ± 81.90^x^	1426.81 ± 63.07^x^	1416.50 ± 74.61^x^
	Female	1135.37 ± 62.63^y^	1166.31 ± 59.99^y^	1148.12 ± 49.44^y^	1149.25 ± 45.52^y^
	Total	1278.94 ± 156.46	1292.12 ± 146.04	1287.47 ± 152.15	1282.87 ± 148.75
Hot carcass weight (%)	Male	79.23 ± 1.14	78.66 ± 0.99	78.76 ± 1.37	78.72 ± 1.18
	Female	78.27 ± 0.96	77.85 ± 0.97	78.68 ± 2.94	77.73 ± 1.47
	Total	78.75 ± 1.15	78.26 ± 1.05	78.72 ± 2.26	78.23 ± 1.41

^x,y^Values with different superscript letters in a row are significantly different (p-value < 0.05).

### Chicken meat characteristics

The quality traits of breast meat of crossbred Pradu Hang Dam chickens obtained from the four treatment groups are shown in Table 6. There was no significant difference in drip loss percentage between the four groups studied in male or female chickens. Although the boiling loss percentages of female chickens in the T30 group were lower than those of the other three groups studied (p-value < 0.05), the combined values were not significantly different (p-value > 0.05).

**Table 6:** Meat quality of breast muscle in crossbred Pradu Hang Dam Chicken obtained from 4 treatment groups.

Items	Gender	Treatments (mean ± SD)			
		0 s	10 s	20 s	30 s
Water holding capacity (WHC)					
Drip loss (%)	Male	0.94 ± 0.34	0.92 ± 0.11	0.79 ± 0.26	1.00 ± 0.22
	Female	1.02 ± 0.39	1.04 ± 0.22	1.16 ± 0.50	1.07 ± 0.14
	Total	0.98 ± 0.35	0.98 ± 0.18	0.98 ± 0.42	1.03 ± 0.17
Boiling loss (%)	Male	14.67 ± 3.26	15.94 ± 1.84	14.02 ± 1.76	14.32 ± 1.47
	Female	16.78 ± 1.52^a^	16.66 ± 1.33^a^	16.61 ± 1.65^a^	13.95 ± 0.94^b^
	Total	15.73 ± 2.61	16.30 ± 1.53	15.32 ± 2.10	14.14 ± 1.16
pH					
	Male	5.65 ± 0.10	5.70 ± 0.09	5.69 ± 0.11	5.76 ± 0.07
	Female	5.65 ± 0.04	5.64 ± 0.12	5.70 ± 0.04	5.66 ± 0.02
	Total	5.65 ± 0.07	5.67 ± 0.11	5.70 ± 0.08	5.71 ± 0.07
Color					
L*	Male	60.27 ± 4.21	58.91 ± 1.33	59.64 ± 2.68	60.03 ± 0.47
	Female	60.99 ± 1.88	61.58 ± 4.02	60.26 ± 4.13	60.59 ± 1.97
	Total	60.63 ± 3.04	60.24 ± 3.12	59.95 ± 3.24	60.31 ± 1.36
a*	Male	1.00 ± 0.67	0.94 ± 0.50	0.84 ± 0.35	1.27 ± 0.93
	Female	1.38 ± 0.43	1.14 ± 0.37	0.91 ± 0.55	1.11 ± 0.31
	Total	1.19 ± 0.56	1.04 ± 0.42	0.88 ± 0.43	1.19 ± 0.65
b*	Male	7.38 ± 1.26	6.73 ± 1.62	6.99 ± 0.57	7.59 ± 1.83
	Female	8.79 ± 0.68	8.27 ± 0.80	7.81 ± 1.23	8.58 ± 0.61
	Total	8.08 ± 1.20	7.50 ± 1.44	7.40 ± 0.99	8.08 ± 1.37

^a,b^Values with different superscript letters in a row are significantly different (p-value < 0.05).

**Figure 3:**
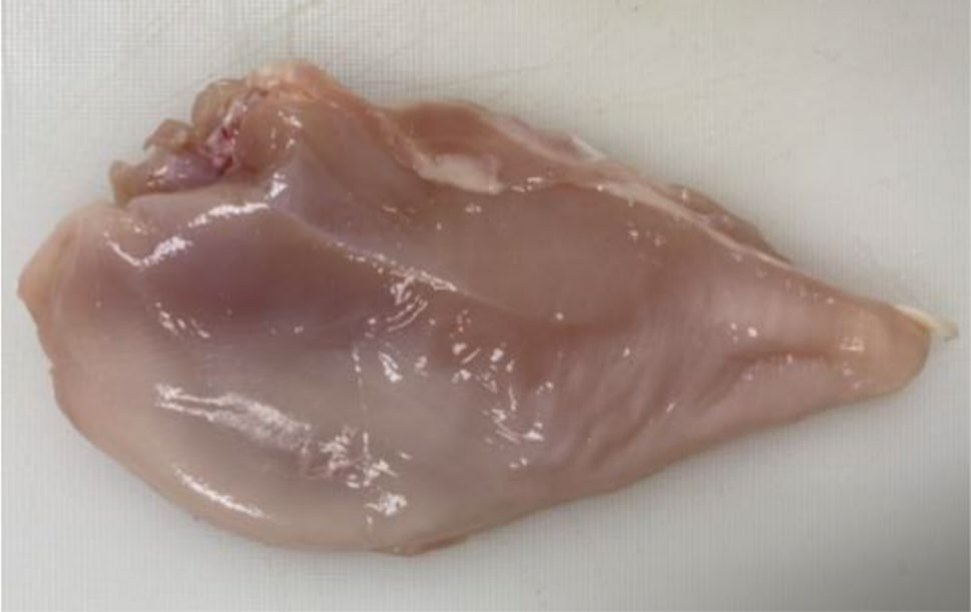
Chicken breast meat after treated with cold plasma technology.

The pH level and the L*, a*, and b*color traits (p-value > 0.05) (Fig. 3) of the cold plasma-treated groups were compared to that of the control group. The color and pH results were in line with those reported by Wang et al. (2018) and Moutiq et al. (2020). They reported insignificant differences in color parameters and pH values after the meat sample was treated with cold plasma at a voltage of 80 V for 3, 6, and 9 min and 100 kV for 5 min on the in-package chicken meat. Cold plasma treatment did not affect the pH of meat samples.

Recently, some reports have shown that the drip loss and pH of chicken breast meat can be slightly affected by cold plasma treatment as treatment time ranged from 60 to 300 s with the voltage ranging from 60 to 80 kV. The color change might be the combined result of long plasma exposure time and the low oxygen content. L*, a*, and b* are affected by various factors, including the treatment time, voltage, and oxygen level^41^. The variation in results between previous studies and this study could result from the different aims of the studies. We conducted the plasma treatment at the hatching egg stage. In contrast, previous studies applied the plasma directly to raw packaged meat. Additionally, the time and voltage parameters varied among studies.

Table. 7 shows the effect of different cold plasma treatment conditions on the shear force in chicken breasts. The cold plasma treatment notably increased the shear force value in breast samples of male chickens with the highest value in T10 (p-value < 0.05). However, it did not significantly differ in female breast samples. This may be because protein oxidation in living tissue targets reactive oxygen species (ROS) caused by cold plasma treatment, which may have induced cross-linking of proteins, including myofibrillar protein in muscle^42^. ROS, such as ozone, OH, H_2_O_2_, O^−^_2_, and O^−^_3_, have short half-lives but are highly responsive. ROS can interact with the aromatic ring of the amino acid side chain of proteins in the muscles^43^. Thus, the potential modification of muscle proteins induced by cold plasma may affect the altered tenderness of the chicken breast. However, the results of the treatment groups (T20 and T30), which were induced by cold plasma under the conditions used in this study, did not bring about significant changes in shear force and texture profile compared to the control (p-value > 0.05).

**Table 7:** Effects of cold plasma on shear force and TPA of boiled chicken breast.

Items	Gender	(Mean ± SD)			
		0 s	10 s	20 s	30 s
Shear force (g)	Male	2499.6 ± 1052.3^b^	4134.9 ± 566.15^a,x^	2945.8 ± 422.33^b^	2938.2 ± 667.29^b^
	Female	3082.0 ± 405.26	2954.6 ± 763.65^y^	3003.3 ± 939.05	2750.4 ± 316.05
Hardness (g)	Male	1033.2 ± 413.28^a,b,y^	1259.3 ± 404.98^a^	847.4 ± 271.61^b,y^	1079.2 ± 522.09^a,b^
	Female	1366.0 ± 417.21^x^	1067.4 ± 389.86	1477.2 ± 449.00^x^	1345.9 ± 669.43
Gumminess (g)	Male	657.9 ± 287.97^a,b,y^	790.56 ± 306.94^a^	521.6 ± 187.51^b,y^	670.3 ± 367.19^a,b^
	Female	898.2 ± 308.18^x^	693.0 ± 267.86	923.9 ± 322.00^x^	842.5 ± 459.30
Chewiness (g)	Male	443.6 ± 192.67^a,b^	514.9 ± 227.72^a^	333.6 ± 130.39^b,y^	428.5 ± 234.29^a,b^
	Female	570.3 ± 193.08	457.2 ± 175.58	632.2 ± 217.50^x^	563.4 ± 325.69

^a,b,c^, and ^x,y^ Values with different superscript letters in a column and row, respectively, are significantly different (p-value < 0.05).

Cold plasma technology was recently used to decontaminate microorganisms in raw chicken meat. A previous study showed that this novel technology did not affect the meat quality traits, such as color, pH, and tenderness^44^.

The TPA results for the chicken breasts are shown in Table 7. Treatment with cold plasma for 10, 20, and 30 s did not significantly change the hardness, gumminess, and chewiness in male or female chickens compared to the control (p-value > 0.05). These results were confirmed by Kang, et al.^45^, who reported that treatment with plasma-activated sterile distilled water for 60 s caused no significant changes in the texture characteristics of chicken breasts. In contrast, the results indicated that cold plasma improved the hardness, gumminess, and chewiness of female breast chicken, higher than those of male breast samples (p-value < 0.05).

In addition, the results showed that the meat characteristics among the studied treatments did not differ in any quality trait when male and female chicken breasts were grouped (p-value > 0.05) (see Supplementary Table S1). The consumers’ requirements for native chicken texture is for tougher meat than that of the commercial chicken breeds. Cold plasma conditions used in this study did not alter meat quality, which is potentially important for consumer satisfaction. However, this study focuses on improving the meat quality of Thai native chicken for commercial products, which involves quality measures that are thought to affect consumer perception, such as physical quality, color, pH, and texture (shear force). Therefore, the study of structural change mechanisms could have been performed by SEM or TEM on muscle cells; this needs to be investigated in future studies.

## Methods

### Preparation of crossbred Pradu Hang Dam Chicken

Fresh Pradu Hang Dam fertilized eggs were obtained from crossbred hens from the commercial layer of Pradu Hang Dam and incubated at the Feed Research and Innovation Center 3 (Chonburi, Thailand). Fertilized eggs weighing 54–56 g were separated into control and treatment groups. In total, 1056 fertilized eggs were incubated in a hatchery incubator for 21 days at 38 °C with 65% relative humidity and rotated 45° every hour. Animal handling protocols were approved by the Institutional Animal Care and Use Committee of the Feed Research and Innovation Center (protocol review number: FRIC-ACUP-2008005). All methods were performed in accordance with the relevant guidelines and regulations. The study was reported in accordance with the ARRIVE guidelines (https://arriveguidelines.org).

### Plasma treatment

**Figure 4:**
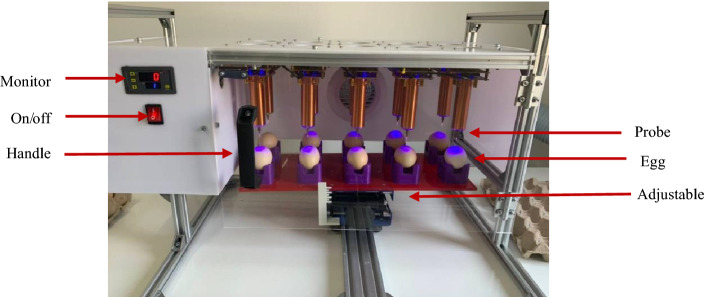
Set-up of 5 × 2 array transient spark plasma device and egg samples holder.

Ambient air radicals were generated using transient spark (TS) plasma in a 5 × 2 array in a semi-closure frame, as shown in Fig. 4. Briefly, the custom-made plasma array device consisted of 10 TS plasma units fixed at the top of a 120 cm × 80 cm aluminum frame. Each TS plasma unit has a sharpened copper electrode, working as a high-voltage electrode for a 20 W RF self-resonance power supply. The power supply can provide a sinusoidal AC output with a peak voltage (Vp) of 6–10 kV at a frequency of 700–900 kHz. In this study, different exposure times (seconds) [0 (T0), 10 (T10), 20 (T20), and 30 (T30)] were employed to generate appropriate doses of reactive species to different extents. Fertilized eggs were subjected to analyses at designated time points (0, 15, and 30 s).

Reactive oxygen species (ROS) and reactive nitrogen species (RNS), e.g., ozone, hydrogen peroxide (H_2_O_2_), and nitrite, are generated in the gas phase during TS plasma production^46^. In this study, the reactive oxygen and nitrogen species H_2_O_2_ and nitrate were analyzed in the aqueous using Mquant^®^ Peroxide and Nitrate Test strips (Merck, Darmstadt, Germany).

### Optimization of plasma treatment condition

Appropriate conditions for the plasma treatment of eggs before hatching were determined. The temperature probe was inserted into the egg to measure the temperature change before and after plasma treatment. The plasma exposure time and distance between the probe and base were varied to evaluate the change in the temperature of the liquid inside the egg. Consequently, the plasma treatment conditions listed in Table 8 were studied. The eggs used in this experiment weighed 63–64 g.

**Table 8:** Plasma treatment condition.

Condition	The distance between probe and base (cm)	Plasma exposure time (s)
1	7	30
2	7	60
3	9	30
4	9	60

### Egg evaluation

Fertilized eggs with and without plasma treatments were incubated for 21 days. Candling was performed on day 18 during the incubation and moved to a hatchery incubator for three days. On the hatching day, breakout analysis involves sampling unhatched eggs and classifying them into various causes of reproductive or incubation condition failure.

### Growth monitoring of Crossbred Pradu Hang Dan chickens

Thirteen chicks from each group were separated by sex and housed in individual cages under natural environmental conditions (28 °C). All were given free access to water and feed containing 16–21% crude protein and 2800 kcal/kg. Chicken weight and shank length were measured on days 0, 21, 42, and 63. Chicken performance indices, including feed intake, average daily gain (ADG), feed conversion ratio (FCR), and survival rate, were calculated. Moreover, to evaluate feeding economics and the efficiency of implementation in the industrial sector, return over feed cost (ROFC) was estimated by Eq. (1).

1 $${\text{ROFC}} = \, ({\text{Chicken}}\,{\text{weight}} \; ({\text{kg}}) \times {\text{ Selling}}\,{\text{price }}({\text{kg}})) \, {-}{\text{Feed}}\,{\text{cost }}({\text{baht}}/{\text{chicken}}).$$

### Electrochemiluminescence immunoassay (ECLIA)

Blood samples from 21-, 42-, and 63-day-old chickens were collected within 1 h. These samples were centrifuged for 5 min at 4000 rpm, and the serum was collected. The somatotropin hormone levels (ng/ml) in the serum of male and female chickens were detected using an electrochemiluminescence immunoassay (ECLIA) according to the manufacturer’s instructions on the Cobas E602 Immunology analyzer (Roche, IN, USA). The detection limit is 0.03 ng/ml.

### Chicken carcass information

On day 63, male and female chickens in each group (n = 128) were slaughtered after 8 h of feed withdrawal. After evisceration and offal weighing, whole carcasses were stored at 4 °C for 1 h until cut and deboned for meat quality evaluation. The carcass ratio was calculated by dividing the carcass weight after offal removal by the weight before slaughter.

### Meat quality evaluation

#### Color

The color of breast meat (pectoralis major) was measured on the medial side of each muscle using a Minolta chromameter (CR-10 Plus; Konica Minolta, Osaka, Japan). Three random points were measured for each sample, and the average value was recorded. Based on CIE color system, three color measurements are represent in the L* a* b* color scale, where L* represents the level of lightness (black to white as shown in the number range of 0–100), a* represents the greenness (− a*) and the redness (+ a*), and b* represents the color of blue (− b*) and yellow (+ b*). The values for each color parameter were averaged.

#### pH value of meat

The pH value of the breast meat was measured 24 h after slaughter using a pH meter (HI99163, Hanna) by inserting the glass electrode into the center of the breast muscle.

#### Weight loss

Breast samples were weighed and packed in plastic bags. The samples were hung on a hook and stored in a refrigerator at 4 °C for 24 h. After storage, the samples were weighed to calculate weight loss.

#### Cooking loss

Cooking loss was performed according to Kondaiah et al. (1985). The cooking loss was measured by heating the breast sample in a water bath. 20 g of sample was placed in a polyethylene bag and heated at 80 °C until the sample reached an internal temperature of 72 °C. The temperature was measured using a thermometer inserted into the center of the meat sample. The exudate was drained from the sample, and the weight loss of meat was calculated using the following Eq. (2).

2 $${\text{Weight}}\,{\text{loss}} = \, ({\text{The}}\,{\text{initial}}\,{\text{sample}}\,{\text{weight}}{-}{\text{the}}\,{\text{final}}\,{\text{sample}}\,{\text{weight}}/{\text{the}}\,{\text{initial}}\,{\text{sample}}\,{\text{weight}}) \times { 1}00\% .$$

#### Shear force

The shear force was measured at room temperature using a texture analyzer (TA-XT plus, Stable Micro System Ltd.). All boiled breast samples were cut to dimensions of 10 mm × 10 mm × 30 mm. The sample was then cut using a Warner–Bratzler shear fixture (V-shaped blade) with 50 kg loading cell, pre-test speed of 5 mm/s, test speed of 1.5 mm/s, and post-test speed of 10 mm/s. The expression report results are shown as the shear force (g).

#### Texture profile analysis

Texture profile analysis (TPA) was performed at room temperature using a texture analyzer (TA-XT plus Model, Stable Micro System) equipped with a 50 kg load cell. All boiled breast samples were cut into cubes (10 mm × 10 mm × 10 mm), double-cycle compressed with 50% deformation using the pre-and post-test speed of 2 mm/s of a 36 mm diameter probe (P/36). Twenty cube samples of each sex were measured for each treatment to obtain an average value for TPA parameters (hardness, cohesiveness, springiness, chewiness, gumminess, and resilience).

### Statistical analysis

Data are presented as the mean ± standard deviation (SD). Statistical analyses were performed using the Statistical Package for the Social Science (SPSS version 26.0). One-way ANOVA determined statistically significant differences with a Duncan’s Multiple Range Test. Values were considered significantly different at p-value < 0.05.

### Approval for animal experiments

All experiments were performed in accordance with Animal handling protocols by the Institutional Animal Care and Use Committee of the Feed Research and Innovation Center (protocol review number: FRIC-ACUP-2008005).

## Conclusions

Crossbred Pradu Hang Dam chicken, consumed as poultry, is one of Thailand’s most famous native chicken breeds. To increase the production and growth rates of the chickens, cold plasma technology was applied to fertilized eggs. Appropriate plasma conditions were determined by varying the input parameters, including the distance between probe and base and plasma exposure time. Moreover, the hatch of fertile (HoF) values and meat quality after plasma treatment were evaluated in this study.

The conditions used in this experiment were a 9 cm distance between probe and base and a plasma exposure time of 30 s and less. This did not cause burn marks on eggshells or protein denaturation in white eggs. The results showed that the average HoF and number of DOCs were not significantly different from those in the control group. Additionally, the DOC body weight between the treatments was not significantly different, but the shank length of female DOC in T20 was significantly higher than that of males. In contrast, the shank length was greater in males than in females at 21, 42, and 63 days. In addition, the chicken performance indices, including feed intake, average daily gain (ADG), and feed conversion ratio (FCR) of females, were not significantly different at every growth stage until 63 days of age. However, the male performance indices of ADG and FCR at 42 and 63 days were significantly different among the treatments. These results indicate that cold plasma technology has a greater impact on male chickens than female chickens. In addition, both males and females in T20 showed the highest return over feed cost, indicating that cold plasma technology can increase profit in the poultry Industry.

In general, consumers prefer smaller-sized native chickens as the meat from the larger sizes will be tougher. The results demonstrated that the weights of slaughtered males and females were similar among treatments and related to customer requirements. There were no significant differences in the quality parameters, including drip loss, boiling loss, pH, and color among the four treatment groups in male or female chickens. Additionally, cold plasma treatment did not significantly affect the hardness, gumminess, and chewiness, which are prominent characteristics of native chickens. Although this technology benefits the production rate, growth rate, and cost reduction and does not significantly affect the breast meat quality, the implementation of cold plasma technology in different poultry industries will use different treatment conditions. Therefore, this study suggests using novel technology to improve production and growth rates in other valuable poultry industries.

## Supplementary Information

Supplementary Table S1.

## Data Availability

The datasets generated during analyzed during the current study are available from C.S. (the corresponding author) on reasonable request.
