# Supplementary material for: Improving the efficiency of crossbred Pradu Hang Dam chicken production for meat consumption using cold plasma technology on eggs
Source: Sci Rep. 2023 Feb 17;13:2836. doi: 10.1038/s41598-023-29471-6 (PMC9938122; doi:10.1038/s41598-023-29471-6)
Supplement: Supplementary file 1 — Supplementary Table S1. [file 41598_2023_29471_MOESM1_ESM.docx]

**Improving the Efficiency of Crossbred Pradu Hang Dam Chicken Production for Meat Consumption using Cold Plasma Technology on Eggs**

Apichaya Sakulthai, Choncharoen Sawangrat*, Duangporn Pichpol, Jutamart Kongkapan, Tiranun Srikanchai, Rangsun Charoensook, Phanumas Sojithamporn, and Dheerawan Boonyawan

**Supplementary Information**

**Supplementary Table. S1.** Effects of cold plasma on shear force and TPA of mixed sex boiled chicken breast.

| **Items** | **(Mean ± SD)** | | | |
| --- | --- | --- | --- | --- |
|  | **0 sec** | **10 sec** | **20 sec** | **30 sec** |
| Shear force (g) | 2,790.8±801.17 | 3,544.7±886.21 | 2,974.6±674.77 | 2,844.3±493.68 |
| Hardness (g) | 1,199.63±443.19 | 1,163.38±404.21 | 1,162.33±485.66 | 1,212.54±607.75 |
| Gumminess (g) | 778.02±318.55 | 741.77±288.61 | 722.74±330.36 | 756.41±419.59 |
| Chewiness (g) | 506.93±200.91 | 486.06±202.82 | 482.87±232.78 | 495.91±288.24 |

*a,b,c, and x,y Values with different superscript letters in a column and row, respectively, are significantly different (p-value<0.05)
